# A stomata-inspired superhydrophobic portable filter system†

**DOI:** 10.1039/d1ra03297f

**Published:** 2021-05-25

**Authors:** Yuanping Ma, Feng Zhao, Lei Wang, Yuchen Ding, Hongbin Zhao, Hao Wang, Jing Liu

**Affiliations:** Department of Stomatology, Beijing Tian Tan Hospital, Capital Medical University Beijing 100070 China; Specialized Robot Engineering and Technological Center of Hainan Province, Hainan Vocational University of Science and Technology Haikou 571126 China; Beijing Key Lab of Cryo-biomedical Engineering, Key Lab of Cryogenics, Technical Institute of Physics and Chemistry, Chinese Academy of Sciences Beijing 100190 China leiwang@mail.ipc.ac.cn; Department of Physics and Astrophysics, Rensselaer Polytechnic Institute Troy 12180 USA; State Key Laboratory of Advanced Materials for Smart Sensing, General Research Institute for Nonferrous Metals Beijing 100088 China; Department of Biomedical Engineering, School of Medicine, Tsinghua University Beijing 100084 China

## Abstract

Stomata, specialized functional openings distributed on the leaf surface, are used for plant respiration by allowing gas exchange, *i.e.*, taking in carbon dioxide and releasing oxygen, and for water content regulation. Their function is vital to plant survival. Leaves with different wettability exhibit different stomata densities. In this study, we find that stomata on *Pistia stratiotes* L. leaves are protected by superhydrophobic setae, which prevent direct contact between the stomata and water in humid environments by suspending water droplets on the top of the setae. Thus, oxygen and carbon dioxide are freely exchanged through the stomata. This structure inspired us to design and develop a mask for filtering solid particles and noxious gas from the atmosphere. The incoming gas is in convective contact with water, achieving a filtering efficiency. The solid particles and potential harmful gas in air are wetted and captured by water, leaving fresh air for healthy breathing. This novel design has potential applications in the treatment of respiratory diseases.

## Abstract 2

The bio-fabricated superhydrophobic filter system (a–c). The liquid water is suspended on the top of the papillae surface and hardly contacts the stomata, which filter the incoming air (d).

## Graphical abstract

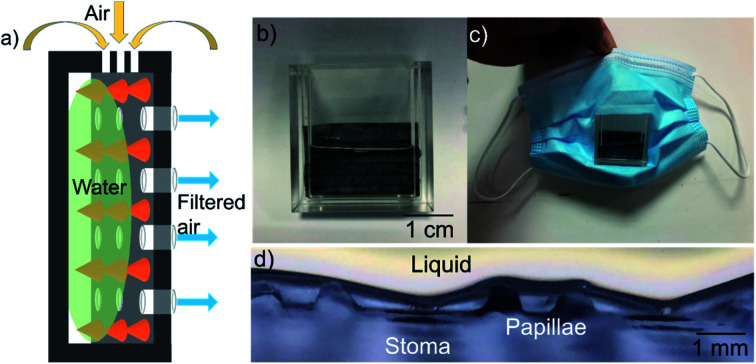

A stoma is a plant gas exchange channel that is used for carbon dioxide intake and oxygen release, playing a crucial role in plant survival in nature.^1–3^ The distribution and density of stomata on the leaf surface may vary with surface wettability. On the hydrophilic leaves of a locust tree, the stomata density on the lower surface is higher than that on the upper surface. This unique design is beneficial for respiration and photosynthesis, even in rainy conditions. Some leaves with superhydrophobic surfaces exhibit a higher stomata density on the upper surface than that on the lower surface, because of their robust water-repellence.^4–7^ Rough micro-/nano-topography may allow significantly more air flow between solid–liquid interfaces with water droplets' suspension on the peak region of the rough solid surface.^8–11^ Thus, the superhydrophobic topography suppresses direct contact between the stomata and water, preventing contamination and yielding healthy plants.

Many plants in nature possess superhydrophobic leaves, *e.g.*, lotus, taro, and *Pistia stratiotes* L.^12–14^ Different from other superhydrophobic plants with micro/nano-topography, *Pistia stratiotes* L. plants have millimetre-level superhydrophobic setae. Their high topographical features are especially beneficial for long-lasting and steady breathing in the water environment. Inspired by this natural design, we developed a novel air filtration system. It consists of stomata arrays integrated with superhydrophobic micro-papillae. The micro-hole arrays filter micro-sized solid particles from the air and provide fresh air for breathing.

*Pistia stratiotes* L. is a self-cleaning floating herbaceous plant that lives on the water surface. The plant's surface is covered with superhydrophobic millimeter-sized setae (mm-setae) arrays for keeping a stable respiration shown in Fig. 1a and b, enabling the plant survival in high-humidity environments. Water droplets with a large surface tension are easily suspended on the surface of setae arrays (Fig. 1c and Movie Online Resource 1†). Thus, they rarely contact the stomata under the setae arrays, maintaining a layer of flowing air at the solid–liquid interface, further enhancing the leaf survivability. Even when the leaf surface is 1 m below the water level, the leaf can survive for more than 24 h due to the amount of air retained between the mm-setae. The ability of the *Pistia stratiotes* L. leaf to survive underwater is better than that of the lotus leaf.^5,15^ In addition, solid dust may be easily captured by a water droplet, and once it is wetted, it cannot get out of the water droplet due to the large surface tension and excellent wettability of water.^16–20^ Fig. S1 and Movie Online Resource 2† show a captured solid particle that floats in the water droplet. In this process, water filters the air.^21^

**Fig. 1:**
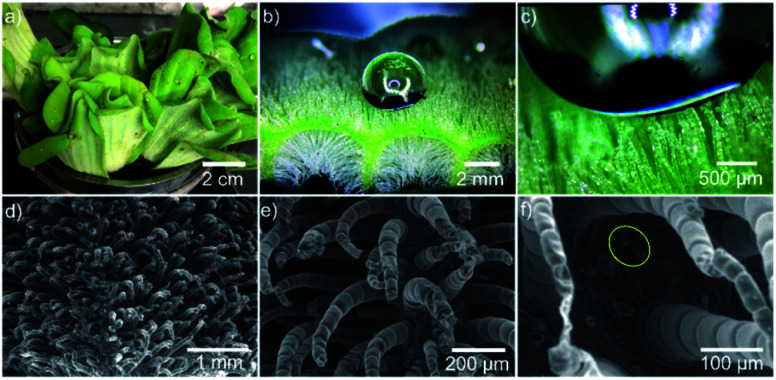
The state of a droplet on the surface of *Pistia stratiotes* L. leaf. (a) An optical image of *Pistia stratiotes* L. on the water surface. (b and c) The droplet suspends on the surface of mm-setae arrays. The droplet cannot directly contact the leaf base and cover the stomata, maintaining the respiration function. The mm-setae are distributed not only on the upper side but also on the lower side of the leaf. (d–f) The SEM micrograph of the leaf surface. (d–f) The surface is covered by mm-setae arrays. The setae with a height of 600–1400 μm are distributed on the upper side and lower side of the leaf. The setae are covered by nano-petals and wax material, which induce the superhydrophobic function. (f) The distribution of stomata on the leaf surface. The stomata are located at the bottom region of the micro-setae.

The surface of the *Pistia stratiotes* L. leaf is composed of mm-setae and stomata. The mm-setae and stomata play two vital roles for plant survival, *i.e.*, they provide the airflow and serve as a gateway for gas exchange, respectively. Fig. 1d–f shows the SEM micrographs of the leaf surface for better insight into topographical details. The mm-setae, with a height of ∼1 mm, are covered by nano-petals and wax material for achieving the superhydrophobic function, and the stomata are located in the root region of the mm-setae (Fig. 1f). Large setae effectively hinder direct contact between stomata and water. Droplet suspends on the top region of rough surface and shows Cassie's state.^22,23^ The mm-setae are distributed not only on the upper surface but also on the lower surface of the leaf (Fig. S2†), both playing a significant role in maintaining water-repellence and respiration, further enhancing the survival ability underwater.^24^

The mm-setae on the leaf surface exhibit excellent mechanical properties required to resist the external force exerted by water and avoid wetting. We used a micromechanical balance to test the mechanical properties of mm-setae on the upper and lower surfaces (Fig. 2). A stress of 3.2 × 10–2 N is generated when the mm-setae are bent at a deformation of 1 mm. The relationship between the deformation and the stored elastic energy (*Γ*) is shown in eqn (1):^22^1
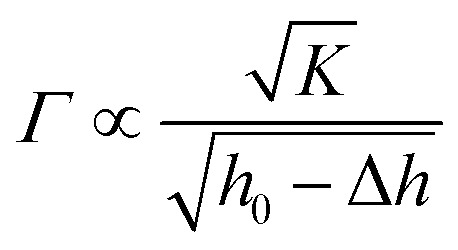
where *K* is the elastic modulus of the mm-setae, *h*_0_ and Δ*h* are the initial height and the deformation of mm-setae, respectively. The bending of the mm-setae becomes challenging as the deformation increase.^25^ The average pressure is larger than 104 Pa, so the water-repellence function is stable even when the structure is soaked in water at a depth of 1 m, making the structure substantially more durable than that of the lotus leaf. As the wetting of stomata is suppressed, the exchange of oxygen and carbon dioxide can take place freely through the stomata even in rainy environment (Fig. 3), resulting in normal respiration. This design improves the vitality and avoids the leaf's degradation.

**Fig. 2:**
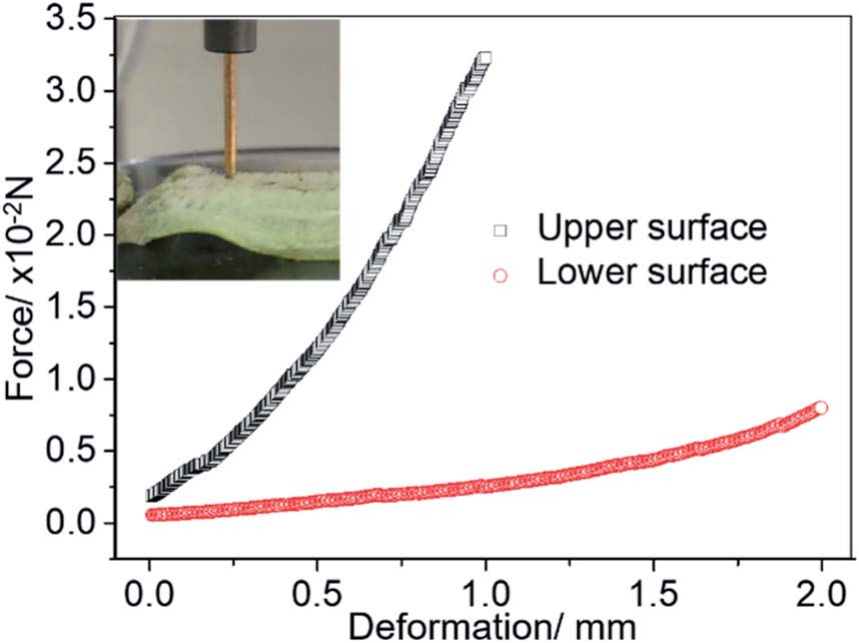
The mechanical test of the mm-setae on the upper and lower surfaces. The diameter of the steel bar is 2 mm. When the mm-setae on the upper surface are bent 1 mm (the line consisting of hollow squares), the average pressure acting on the surface is larger than 1 × 104 Pa, which illustrates that superhydrophobic function still exists 1 m below the water level. The results of mechanical testing indicate the pressure necessary for the water intrusion.

**Fig. 3:**
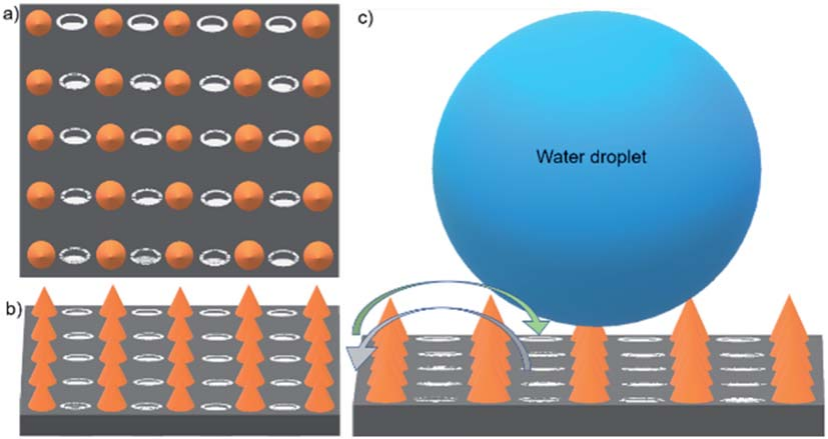
Schematic representation of respiration on the surface of the *Pistia stratiotes* L. leaf. (a and b) The top and side view of the topography. (c) The droplet suspends on the top region of the micro-setae surface. The stomata are located at the bottom of the mm-setae, avoiding direct contact with water.

Inspired by the functionality of the combination of stomata and superhydrophobic mm-setae, we designed a superhydrophobic membrane with upper surface composed of superhydrophobic micro-papillae and micro-stomata arrays (Fig. 4). The micro-papillae, with a height of 500 μm and a base diameter of 500 μm, could provide more air flow and induce a liquid droplet suspension on their tips (Fig. 4b). The contact angle of the 10 μL-droplet is larger than 152°, and the droplet quickly sheds off the surface with a tilting angle of 2°, providing a passage for air (inset in Fig. 4b). The micro-stomata are located between the micro-papillae and extend through the membrane. The long and short diameters of elliptical stomata are 1 mm and 500 μm, respectively. The space between two neighboring stomata, with a width of 500 μm, provides the superhydrophobic function and a lot of air between the solid surface and water. The stomatal channels with a length of 4 mm pass through the whole membrane (Fig. S3†). When the surface is covered by water, the micro-papillae generate small bubbles and air layer at liquid/solid interface (Fig. 4c). The three-phase contact line is very unstable, as it shrinks and detaches from the solid surface when the surface is tilted at an angle of 2° (Fig. 4d). The excellent superhydrophobic performance indues liquid droplet suspension on the tip of micro-papillae, preventing liquid from contacting with the stomata (Fig. 4e and S4†). In the magnified view of the surface shown in Fig. 5, the surface is covered by micro sphere and ZnO nano-rod, which enhances the roughness and further water-repellence function.

**Fig. 4:**
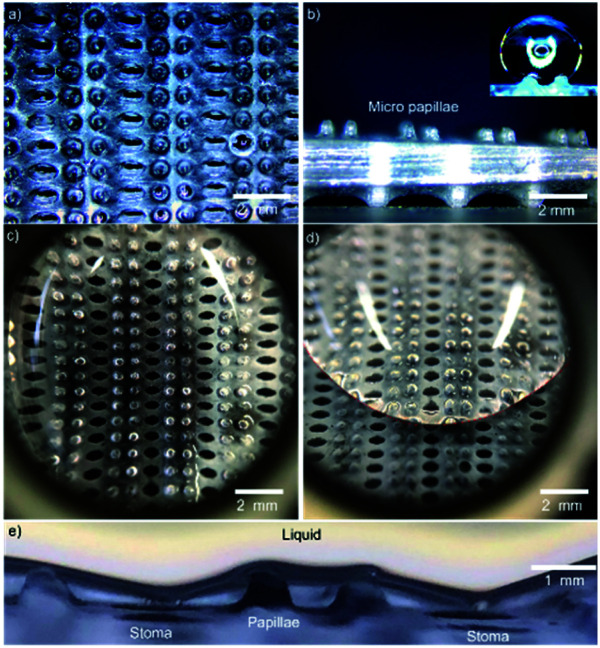
The bio-fabricated gas transmission system. (a and b) The top view and side view of the bio-fabricated surface. The upper surface is composed of superhydrophobic micro-papillae and micro-stomata. The micro-papillae with a height of 500 μm and a base diameter of 500 μm are shown in (b). The inset shows the superhydrophobic state. The droplet suspends on the top of the micro-papillae. The contact angle is larger than 150°. The micro-stomata are located between the micro-papillae and extend through the membrane. (c and d) Top view of the droplet on the bio-fabricated surface. The micro-papillae surfaces generate small bubbles and air layer between the liquid/solid interface. The three-phase contact line (the red curve) is very unstable. (e) The magnified side view of the interface. Liquid suspends on the micro-papillae surface and could not contact the stomata.

**Fig. 5:**
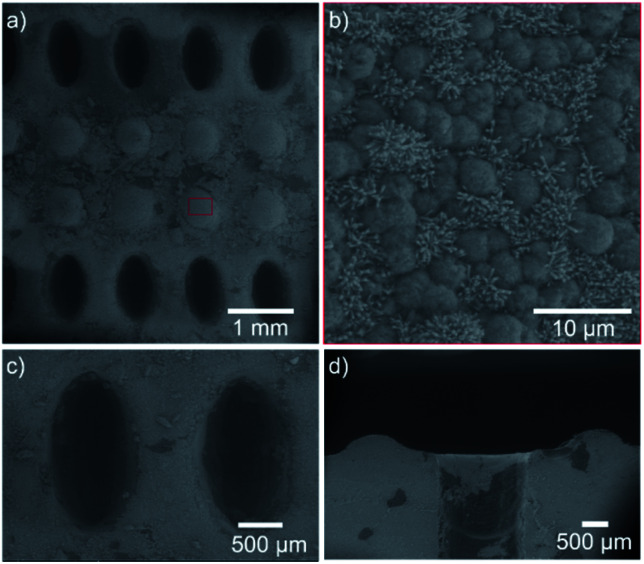
The SEM images of the bio-fabricated membrane. (a) The surface is composed by elliptic micro-stomata array and micro-papillae array. (b) The papilla surface is covered by micro sphere and ZnO nano-rod. The diameter and height of nano-rods are 90∼110 nm and 2 μm, respectively. (c and d) The top view and side view of the elliptic stomata.

The designed bio-fabricated respiratory system is constructed by this membrane equipped with a polymethyl methacrylate (PMMA) shell, and it is used to capture micro-sized solid particles and gas pollutants from the atmosphere (Fig. 6). The membrane is locked in the PMMA shell as a gate to prevent the infiltration of water as shown in the Movie 3.† A filter mask is obtained after mounting the respiratory system on a mask. Fig. 7a shows its working schematic. During the breathing process, the air moves through the valley of the superhydrophobic micro-papillae after entering the respiratory system, and it is filtered by water before being transported into the breathing system (Fig. 7b). Most of solid micro particles and odorous gas once contact the water, they are wetted and absorbed by the water. As the stale gas passes through the sample, it turns into cleaner air. Practical application process by medical staff illustrate that the mask system filters not only solid dusts but also harmful gases in the air (Fig. 7c). Most of the solid particles from the air are absorbed due to their excellent water wettability (Fig. S5†). This design effectively improves respiratory system.

**Fig. 6:**
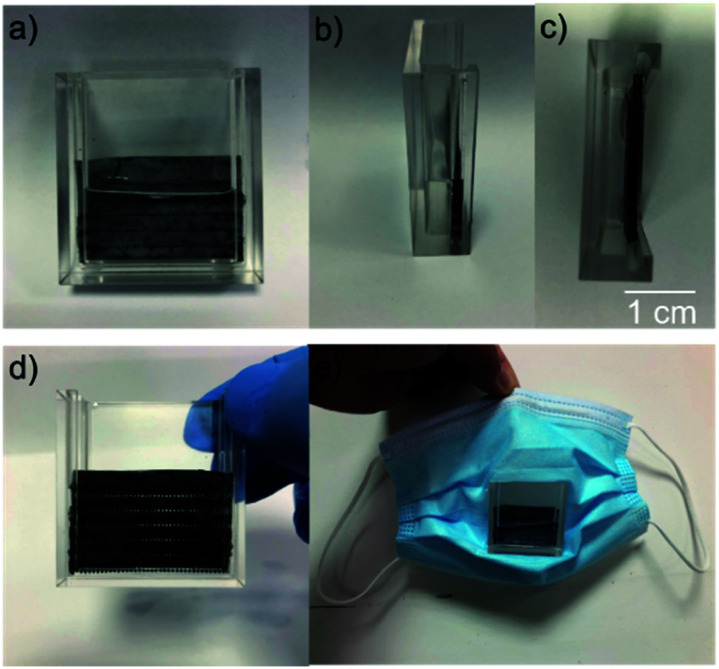
The bio-fabricated respiratory system. (a–d) Different side views of the bio-fabricated system (Movie S3†). (e) The filter system is implemented in a mask.

**Fig. 7:**
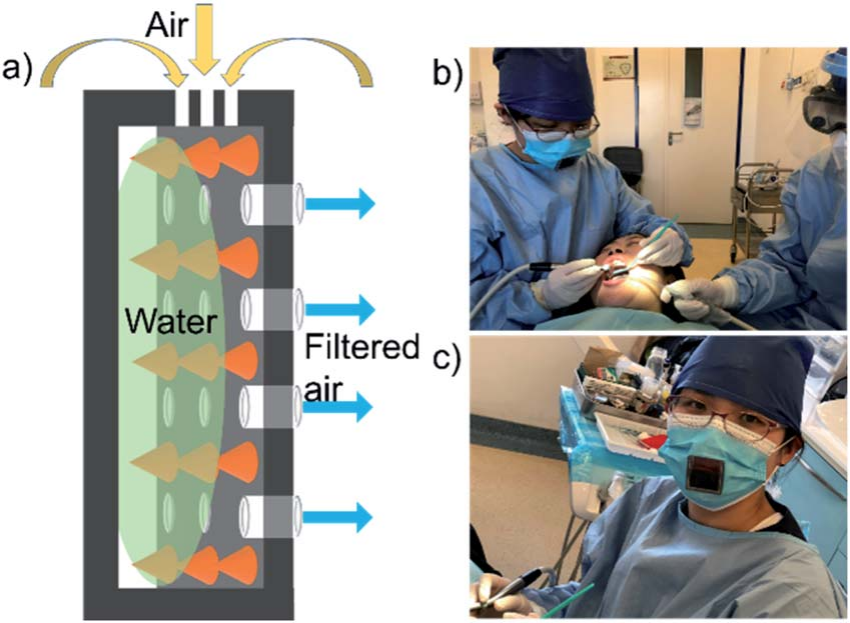
The bio-fabricated respiratory system. (a) Schematic drawing of the system. The bio-fabricated surface is embedded in a groove. The groove is filled with water. The air is filtered by water, so the clean air moves through the stomata. (b and c) Practical application process by medical staff. The mask system not only filter the solid dust in atmosphere, but also harmful gases from the air.

The water in the PMMA shell could be replaced by a volatile drug to treat diseases. Drugs are transported through the stomata and enter the body by the respiratory tract. The size of stomata controls the drug flowing speed. Moreover, this system can be recycled. When the water/drug solution in the PMMA shell is dry, the shell could be reused after washing with water. The number of reuses can exceed 120 times.

In conclusion, inspired by the topography of the natural leaf surface, we developed a novel kind of air filter. The bio-fabricated surface is equipped with superhydrophobic micro-setae and perforating structural micro-stomata. Water suspends on the superhydrophobic micro-setae, providing significantly more flowing air at the valley of the micro-setae. The air is filtered when it goes through the valley and the stomata, providing fresh, comfortable, and moderately humid air for the human respiratory system. This study provides an effective fabrication method for respiratory filtration systems, having potential applications for masks, breather valves, and medical treatment.

## Conflicts of interest

There are no conflicts to declare.

## Supplementary Material

RA-011-D1RA03297F-s001

RA-011-D1RA03297F-s002

RA-011-D1RA03297F-s003

RA-011-D1RA03297F-s004
