# Supplementary material for: A stomata-inspired superhydrophobic portable filter system
Source: RSC Adv. 2021 May 25;11(31):18783–6. doi: 10.1039/d1ra03297f (PMC9033496; doi:10.1039/d1ra03297f)
Supplement: RA-011-D1RA03297F-s004 [file RA-011-D1RA03297F-s004.pdf]

Electronic Supplementary Material (ESI) for RSC Advances.
This journal is © The Royal Society of Chemistry 2021

A Stomata-Inspired Superhydrophobic Portable Filter System

Yuanping Ma[a], Feng Zhao[b], Lei Wang[c], Yuchen Ding[d], Hongbin Zhao[e], Hao Wang[a], Jing Liu[c,f]*

a) Department of stomatology, Beijing Tian Tan Hospital, Capital Medical University, Beijing 100070, China

b) Specialized Robot Engineering and Technological Center of Hainan Province, Hainan Vocational University of Science and Technology, Haikou 571126, China

c) Beijing Key Lab of Cryo-biomedical Engineering and Key Lab of Cryogenics, Technical Institute of Physics and Chemistry, Chinese Academy of Sciences, Beijing 100190, China
**E-mail: leiwang@mail.ipc.ac.cn*

d) Department of Physics and Astrophysics, Rensselaer Polytechnic Institute, Troy 12180, U.S.

e) State Key Laboratory of Advanced Materials for Smart Sensing, General Research Institute for Nonferrous Metals, Beijing 100088, China

f) Department of Biomedical Engineering, School of Medicine, Tsinghua University, Beijing 100084, China

Experimental Section

Preparation of sample

For membrane fabrication, we integrated 3D printing, soft-lithography, and crystal growth methods. First, the negative model was designed and fabricated by 3D printing. Fig. S6 shows schematic drawings and optical images of the topography of the negative model. The 3D model surface was composed by pillar arrays and hole arrays. Those pillars had a height of 3mm and an ellipse upper surface which had long and short diameters of 1mm and 500 μm. They were used to make stomata in the next fabrication process. The depth and diameter of hole arrays were 500 μm. These holes were used to produce micro-papillae after duplicating. These two structures were arranged alternately on the surface, with a space of 1 mm between two neighbour topographies. Afterwards, we used silicone to duplicate the negative 3D model and obtain positive topography. The silicone and curing material were mixed in a weight ratio of 1:1 and poured onto the 3D model surface. After 24 h at 25 °C, the membrane with papillae and stomata arrays was obtained after peeling from the model surface. Then, the surface was coated with a superhydrophobic coating to decrease the surface energy. The coating was ZnO nanorods grown via an in-situ crystal growth method at 90 °C. We used liquid metal as a seed for the crystal growth on the surface. We melted together three metals to produce liquid metal alloy ($Ga_{88}In_{10}Zn_2$) at 150 °C for 6 h, and obtained the liquid metal after the temperature dropped to room temperature. The solid silicone surface was coated with liquid metal by dip-coating method. The liquid metal layer on the surface was oxidized in atmosphere. The metal oxide as a crystal growth seed was formed after painting. 0.30 g of hexamethylenetetramine and 0.72 g of $Zn(NO_3)2{\cdot}6H_2O$ were mixed into 100 mL of deionized water and reacted in a reactor at 85 °C for 12 h to obtain ZnO nano-rods. Additionally, heptadeca fluorodecyltri-propoxysilane was used to tailor the surface energy of the as-fabricated

samples at 90 ℃ for 5 h (at a vacuum degree of 0.1 MPa).

The designed superhydrophobic membrane is enclosed in a PMMA groove structure. The membrane performed as a gate to lock water inside the PMMA shell. The filter is then equipped with a facemask.

Measurement and characterization

Mechanical property characterization: The mechanical property of mm-setae on the leaf upper surface and lower surface were tested by an improved force balance meter system (DCA21, Dataphysics, Germany). The leaf was fixed by glasses. In the bending process, the deformation and stress data were recorded by software.

Scanning Electron Microscopy: The SEM images of the leaf and membrane were observed by Environmental Scanning Electron Microscopy (ESEM, Quanta FEG 250, FEI) under the voltage of 10 kV and lower vacuum.

Evaluation of filtration effect: Double masks are used to evaluate the filtration effect. The designed product is fixed in the outer layer, and the commercial product is fixed in the inner layer. The double masks were worn for 5 hours by cleaning workers and medical staff. Comparing the residual solids on the surfaces of the two masks by SEM, the filtration ratio of the designed mask to solid particles of different size were observed and recorded.

Supplementary information is included as follows:

Supplementary Figures and Legends.

Supplementary Movies.

Supplementary Figure Legends: (Figure S1-S6)

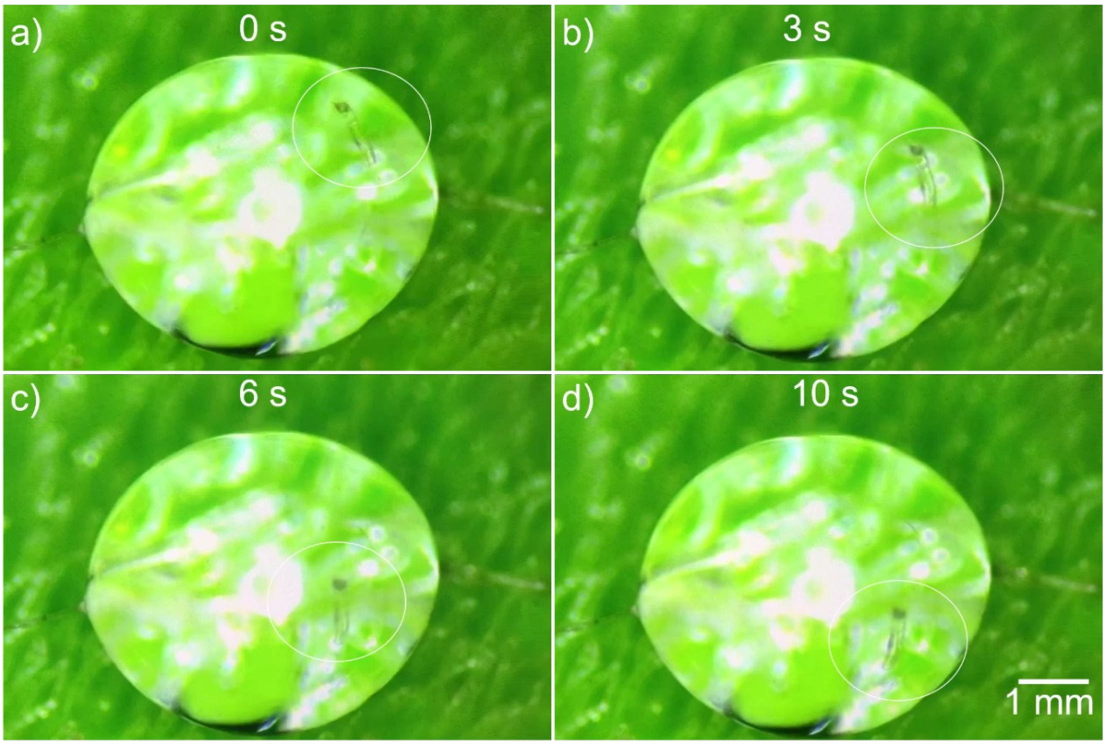

Fig. S1 A solid particle is captured and wetted by a water droplet. The solid particle flows inside the droplet. The air is filtered by the droplet

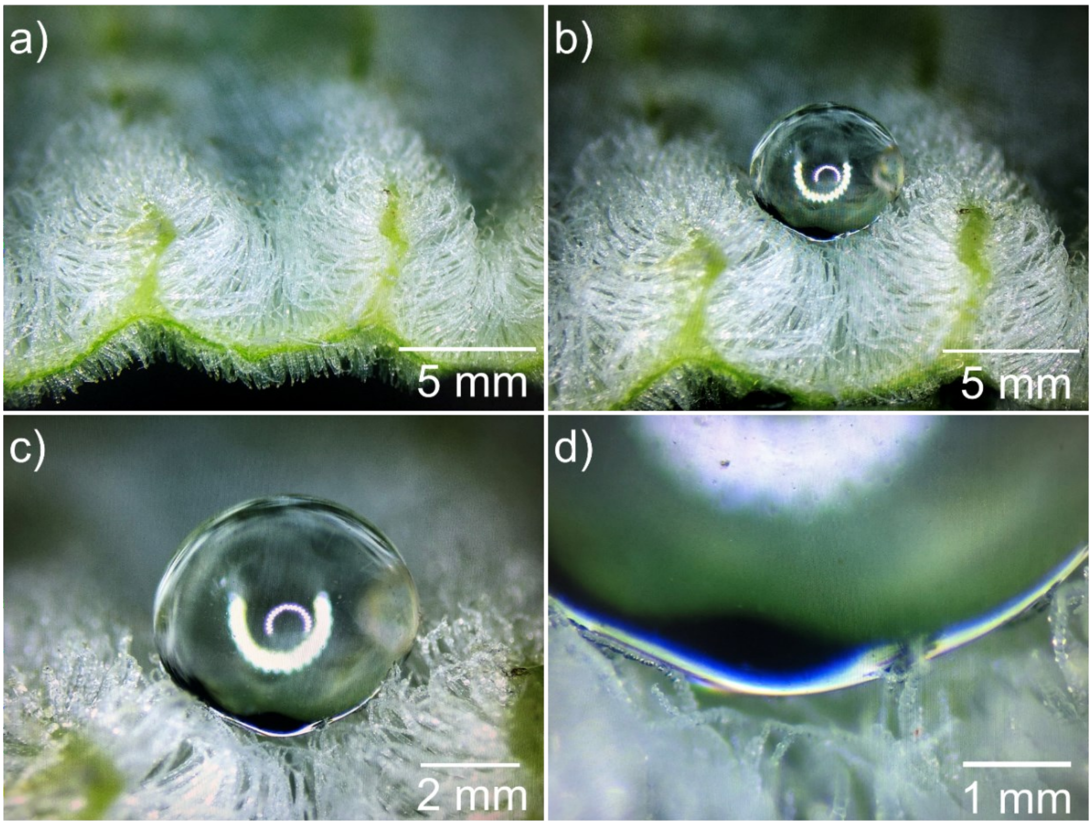

Fig. S2 The state of droplet on the lower surface of leaf. The droplet suspends on the top region of mm-setae

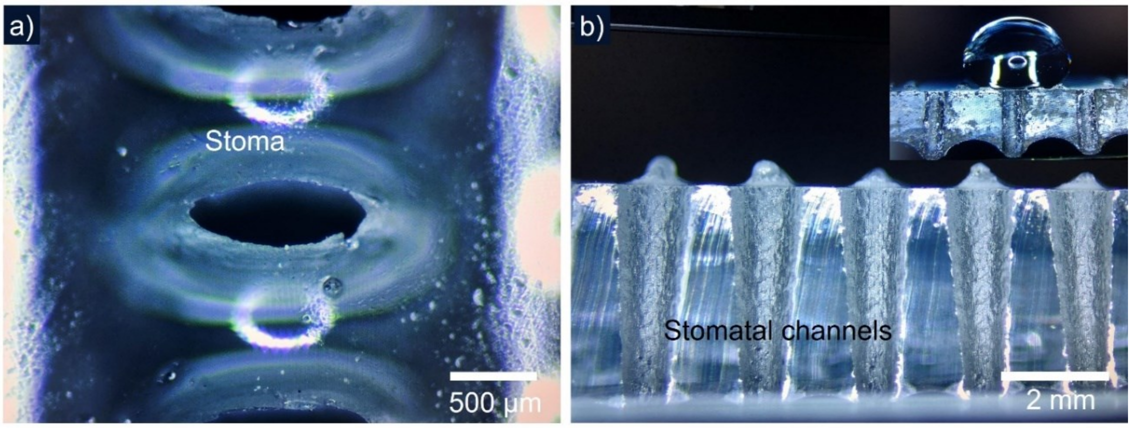

Fig. S3 a) The long and short diameters of elliptical stomata are 1mm and 500 μm, respectively. The space between two neighboring stomata is 500 μm. **b)** The view on the surface from the other side. The stomatal channels have a height of 4 mm. The inset shows the state of the droplet on the surface

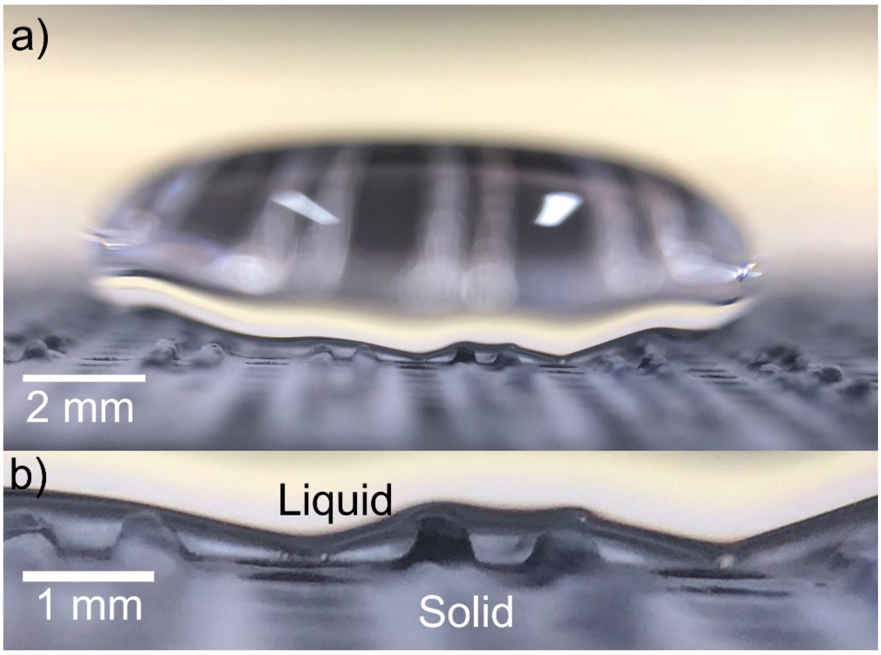

Fig. S4 The side view of the solid/liquid interface of the bio-fabricated surface. The liquid suspends on the top region of micro-setae and hardly directly contact with the stomata

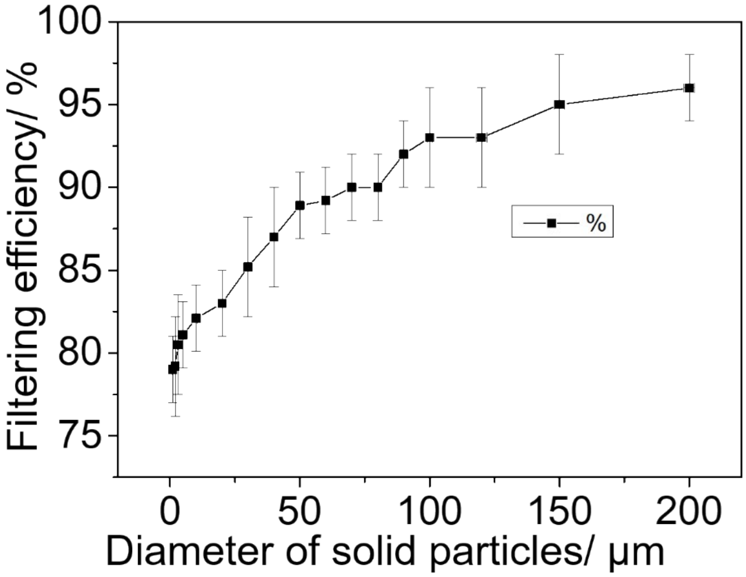

Fig. S5 The filtering efficiency. During the inhalation, solid particles in the air having a larger diameter are easily filtered. The filtering efficiency declines as the particle diameter decreases

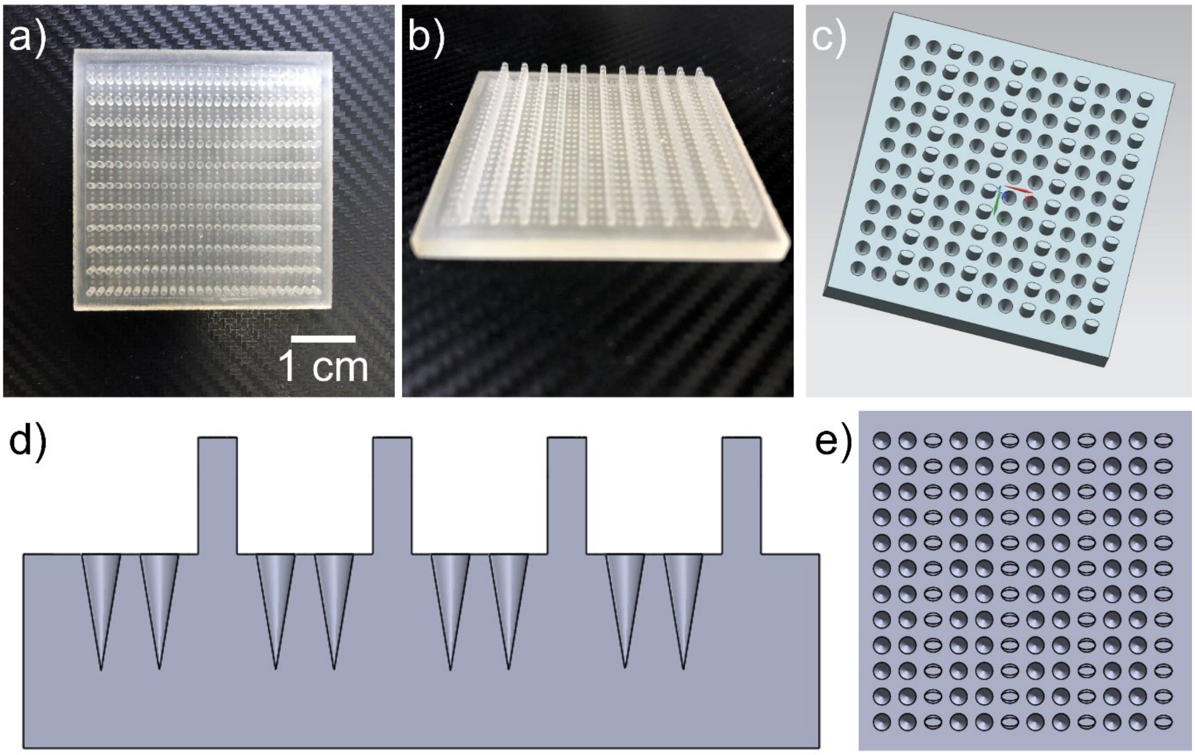

Fig. S6 The model with negative topography. The model is composed by the raised pillar arrays and concave arrays. a-b) The optical images of the model. c-e) The sketches of the model

Supplementary Movies:

Online resource 1: **Movie S1**: State of droplet on the leaf surface.

Online resource 2: **Movie S2**: Self-cleaning performance of the leaf surface.

Online resource 3: **Movie S3**: The process of filling the filter with water.
